# Novel Plastid-Nuclear Genome Combinations Enhance Resistance to Citrus Canker in Cybrid Grapefruit

**DOI:** 10.3389/fpls.2018.01858

**Published:** 2019-01-07

**Authors:** Mayara M. Murata, Ahmad A. Omar, Zhonglin Mou, Christine D. Chase, Jude W. Grosser, James H. Graham

**Affiliations:** ^1^Citrus Research and Education Center, University of Florida, Lake Alfred, FL, United States; ^2^Biochemistry Department, Zagazig University, Zagazig, Egypt; ^3^Department of Microbiology and Cell Science, University of Florida, Gainesville, FL, United States; ^4^Horticultural Sciences Department, University of Florida, Gainesville, FL, United States

**Keywords:** cybridization, plastid, disease resistance, *Xanthomonas citri* subsp. *citri*, *Citrus*

## Abstract

Host disease resistance is the most desirable strategy for control of citrus canker, a disease caused by a gram-negative bacterium *Xanthomonas citri* subsp. *citri*. However, no resistant commercial citrus cultivar has been identified. Cybridization, a somatic hybridization approach that combines the organelle and nuclear genomes from different species, was used to create cybrids between citrus canker resistant ‘Meiwa’ kumquat (*Fortunella crassifolia* Swingle snym. *Citrus japonica* Thunb.) and susceptible grapefruit (*Citrus paradisi* Macfad) cultivars. From these fusions, cybrids with grapefruit nucleus, kumquat mitochondria and kumquat chloroplasts and cybrids with grapefruit nucleus, kumquat mitochondria and grapefruit chloroplasts were generated. These cybrids showed a range of citrus canker response, but all cybrids with kumquat chloroplasts had a significantly lower number of lesions and lower *Xanthomonas citri* subsp. *citri* populations than the grapefruit controls. Cybrids with grapefruit chloroplasts had a significantly higher number of lesions than those with kumquat chloroplasts. To understand the role of chloroplasts in the cybrid disease defense, quantitative PCR was performed on both cybrid types and their parents to examine changes in gene expression during *Xanthomonas citri* subsp. *citri* infection. The results revealed chloroplast influences on nuclear gene expression, since isonuclear cybrids and ‘Marsh’ grapefruit had different gene expression profiles. In addition, only genotypes with kumquat chloroplasts showed an early up-regulation of reactive oxygen species genes upon *Xanthomonas citri* subsp. *citri* infection. These cybrids have the potential to enhance citrus canker resistance in commercial grapefruit orchards. They also serve as models for understanding the contribution of chloroplasts to plant disease response and raise the question of whether other alien chloroplast genotypes would condition similar results.

## Introduction

Citrus canker, caused by *Xanthomonas citri* subsp. *citri*, is a serious disease in terms of economic losses to the citrus industry and is present in most citrus-producing areas of the wet subtropics worldwide (Graham et al., 2004; Pruvost et al., 2014). The financial impact is strongly associated with the biological and epidemiological characteristics of the disease. The causal agent is easily disseminated, and once the bacterium is established in an orchard, the disease can cause defoliation, blemished fruit, premature fruit drop and general tree decline (Gottwald et al., 2002; Graham et al., 2004). Citrus canker is a quarantine disease. In Florida, citrus plants and fruits moving into and out of the state need to be inspected and certified by the USDA (Dewdney and Graham, 2012). Besides these regulatory obstacles, control of citrus canker is costly for growers. In canker-free areas, it is important to prevent bacterial entry into the orchard through decontamination of equipment, vehicles and personnel and to inspect and remove symptomatic trees (Graham et al., 2004; Gottwald et al., 2001). In areas where citrus canker is endemic, use of windbreaks, protection of fruits and leaves with copper bactericidal sprays, control of leaf miner and use of tolerant citrus cultivars are recommended (Graham et al., 2004; Dewdney and Graham, 2012).

The use of host resistance is the most effective way to manage various plant diseases, including citrus canker, but no commercial citrus resistant to this disease has been identified (Deng et al., 2010). Nevertheless, commercial citrus cultivars are not equally susceptible to *Xanthomonas citri* subsp. *citri*. Grapefruits, navel oranges, and some early sweet oranges are considered highly susceptible. ‘Hamlin’ oranges and tangelos are less susceptible, but suffer severe loss of fruit under the most conducive conditions for *Xanthomonas citri* subsp. *citri* infection. ‘Valencia’ oranges, tangerines and tangors are tolerant to citrus canker (Gottwald et al., 2002; Fu et al., 2012). Approaches ranging from conventional breeding methods to production of transgenic plants are being utilized in order to produce resistant plants (Viloria et al., 2004; Grosser et al., 2005; Liu and Deng, 2007; Mendes et al., 2010; Zhang et al., 2010; Machado et al., 2011; Yang et al., 2011; Guo et al., 2013; Omar et al., 2018).

Transgenic citrus plants that express antimicrobial proteins (Boscariol et al., 2006; Stover et al., 2013), harpin proteins (Barbosa-Mendes et al., 2009) and resistance genes from other plant species (Mendes et al., 2010; Zhang et al., 2010; Dutt et al., 2015; Omar et al., 2018) have been produced. Most of these transformed plants have reduced citrus canker severity associated with activation of resistance pathways. However, there is no prediction for when these transgenic plants will be available for growers since many regulatory steps need to be approved until the release of a genetically modified (GM) fruit crop (Potrykus, 2013). Even if the transgenic plants are released, there is still the issue of public acceptance for GM products (Lucht, 2015), and therefore other alternatives should be considered for developing citrus canker resistance in desirable cultivars.

For conventional breeding approaches, the source of resistance comes from citrus germplasm. Some non-commercial citrus and citrus-related species possess field resistance to citrus canker. Calamondin (*Citrus mitis*) and kumquats (*Fortunella* spp.) are considered highly resistant (Khalaf et al., 2007, 2011; Deng et al., 2010). Even though these species are sexually compatible with commercial citrus cultivars, including sweet oranges and grapefruits, few commercial outcomes from these crosses have been released (Viloria et al., 2004). Several factors contribute to the lack of conventional breeding success in citrus, especially those associated with botanical and biological characteristics, such as apomixis, high heterozygosity and a long juvenility period (Grosser et al., 2000; Navarro et al., 2004; Machado et al., 2011). Finally, the lack of genetic knowledge for the inheritance of important horticultural traits makes it difficult to identify a promising cross (Navarro et al., 2004).

By comparison, somatic hybridization has been widely used in citrus breeding because this approach overcomes the main obstacles of conventional breeding (Grosser and Gmitter, 1990; Grosser et al., 2000, 2005). This technique allows production of hybrids with genomes of two parents avoiding the problems associated with heterozygosity and incompatibility (Navarro et al., 2004). Citrus is one of a few commodities where the potential of somatic hybridization has been extensively used for scion and rootstock improvement (Grosser and Gmitter, 1990; Grosser et al., 2000). One interesting outcome from this technique is the production of cybrids by asymmetric protoplast fusion procedures in which the nucleus of one species is combined with the cytoplasm of another species (Moreira et al., 2000; Cai et al., 2009; Bassene et al., 2011; Guo et al., 2013; Omar et al., 2017). Although the precise mechanisms that govern this outcome are unknown, it is likely due to the behavior of organelles as discussed by Greiner et al. (2015). Given a condition of heteroplasmy (more than one organelle genotype in a cell), organelles sort to homogeneity through subsequent cell division. This allows investigation of nuclear-cytoplasmic genome interactions in citrus breeding. Following this approach, protoplast fusions of ‘Meiwa’ kumquat with three different grapefruit cultivars (‘Marsh,’ ‘Flame,’ and ‘N11-11’ somaclone of ‘Ruby Red’ grapefruit) were performed with the goal of producing grapefruit cultivars with the potential for citrus canker resistance. Grapefruit mesophyll protoplasts were used as the nuclear donor and protoplasts from embryogenic suspension culture cells of kumquat served as the cytoplasmic donor. Protoplast somatic fusion mediated by the polyethylene glycol (PEG) method was used to successfully regenerate over 100 diploid grapefruit cybrids (Omar et al., 2017).

Even though the production is not as large as that of oranges, grapefruit is very important for the fresh fruit market and juice industry in Florida. During the 2012–2013 production season, 18 million boxes of grapefruit were harvested (Hodges et al., 2014). However, as mentioned previously, grapefruits are highly susceptible to citrus canker and several disease management strategies are required to successfully produce grapefruit in endemic citrus canker areas (Dewdney and Graham, 2012). Based on this, grapefruit was chosen as a nuclear donor to keep the important horticultural traits, and kumquat was chosen as a cytoplasmic donor due to citrus canker resistance observed in orchards (Khalaf et al., 2007). These new cybrids were produced in an effort to create grapefruit cultivars morphologically equivalent to standard commercial grapefruit but with enhanced citrus canker resistance (Omar et al., 2017). These were genotyped with two plastid and two mitochondrial markers and two different classes of cybrids were identified: one carrying both kumquat organelle genomes (Cybrid-K) and another one with kumquat mitochondria and grapefruit plastid genomes (Cybrid-G) (Omar et al., 2017).

This study aims to characterize the citrus canker response in these two different grapefruit cybrid types and compare their disease phenotypes to those of ‘Meiwa’ kumquat and ‘Flame,’ ‘Marsh,’ and ‘Ruby Red’ grapefruit. These studies will provide insight into the role of cytoplasmic genomes in host–pathogen interactions and identify novel genetic strategies to develop important disease response traits. Moreover, this study may lead to the development of important commercial grapefruit cultivars with resistance to citrus canker.

## Materials and Methods

### Plant Material

The present study used 22 diploid grapefruit cybrids regenerated from protoplast fusions between ‘Meiwa’ kumquat and three grapefruit cultivars ‘Marsh,’ ‘Flame,’ and ‘N11-11’ somaclone of ‘Ruby Red.’ Protoplasts of embryogenic suspension culture of ‘Meiwa’ kumquat were derived from the citrus embryogenic callus collection of the University of Florida’s Citrus Research and Education Center (UF-CREC) and mesophyll protoplasts were isolated from fully expanded leaves of the three grapefruit cultivars. The production and confirmation of these cybrids are fully described in a previous study (Omar et al., 2017).

The regenerated grapefruit cybrids have two different genotypes: 18 Cybrid-K types with grapefruit nucleus, kumquat mitochondria, and kumquat chloroplast and 4 Cybrid-G types with grapefruit nucleus, kumquat mitochondria and grapefruit chloroplast (Table 1). Ten plants from each genotype were T-bud grafted onto US-802 rootstocks, a cross between ‘Siamese’ pummelo (*Citrus grandis*) and ‘Gotha Road’ trifoliate orange (*Poncirus trifoliata*). Plants were cultivated in soilless medium (The Scotts Co., Marysville, OH, United States) contained in 3.8 L pots and maintained in the greenhouse between 20 and 30°C. Plants were fertilized twice a year with slow release fertilizer containing NPK (12–3–9) plus minor elements (Harrell’s LCC, Lakeland, FL, United States). After 1 year, these replicates were moved to a quarantine greenhouse used exclusively for citrus canker inoculation assays.

**Table 1:** List of genotypes used on citrus canker inoculation assays.

Genotypes	Fusion parental genotype		Organelle genome	
		
	Parent A	Parent B	Mitochondria	Chloroplast
**Parents**				
‘Marsh’ grapefruit	–	–	Grapefruit	Grapefruit
‘Flame’ grapefruit	–	–	Grapefruit	Grapefruit
‘N11-11’ grapefruit^∗^	–	–	Grapefruit	Grapefruit
‘Meiwa’ kumquat	–	–	Kumquat	Kumquat
**Cybrids**				
M-9	‘Meiwa’	‘Marsh’	Kumquat	Kumquat
M-10	‘Meiwa’	‘Marsh’	Kumquat	Kumquat
M-11	‘Meiwa’	‘Marsh’	Kumquat	Kumquat
M-13	‘Meiwa’	‘Marsh’	Kumquat	Kumquat
M-31	‘Meiwa’	‘Marsh’	Kumquat	Kumquat
M-78	‘Meiwa’	‘Marsh’	Kumquat	Grapefruit
M-81	‘Meiwa’	‘Marsh’	Kumquat	Grapefruit
M-102	‘Meiwa’	‘Marsh’	Kumquat	Grapefruit
F-2	‘Meiwa’	‘Flame’	Kumquat	Kumquat
F-3	‘Meiwa’	‘Flame’	Kumquat	Kumquat
F-5	‘Meiwa’	‘Flame’	Kumquat	Kumquat
F-6	‘Meiwa’	‘Flame’	Kumquat	Kumquat
F-10	‘Meiwa’	‘Flame’	Kumquat	Kumquat
F-13	‘Meiwa’	‘Flame’	Kumquat	Kumquat
F-15	‘Meiwa’	‘Flame’	Kumquat	Kumquat
F-20	‘Meiwa’	‘Flame’	Kumquat	Kumquat
N-4	‘Meiwa’	‘N11-11’	Kumquat	Grapefruit
N-6	‘Meiwa’	‘N11-11’	Kumquat	Kumquat
N-8	‘Meiwa’	‘N11-11’	Kumquat	Kumquat
N-10	‘Meiwa’	‘N11-11’	Kumquat	Kumquat
N-12	‘Meiwa’	‘N11-11’	Kumquat	Kumquat
N-18	‘Meiwa’	‘N11-11’	Kumquat	Kumquat


*^∗^‘N11-11’ is somaclone of ‘Ruby Red’ grapefruit.*

### Bacterial Strain and Culture Conditions

The *Xanthomonas citri* subsp. *citri* strain 2004-00054 was used for all inoculation assays. This strain was isolated in 2004 from sweet orange (*C. sinensis*) in Dade County, FL, United States. The strain was stored in glycerol under -80°C conditions in an ultra-low freezer. For inoculation assay, the *Xanthomonas citri* subsp. *citri* culture stored in glycerol was thawed and streaked on BD Difco^TM^ Nutrient Agar (0.3% beef extract, 0.5% peptone, 1.5% agar) (Fisher Scientific, Pittsburgh, PA, United States). The plate was kept at 28°C for 48–72 h. A single bacterial colony was seeded into 25 mL of sterile BD Difco^TM^ Nutrient Broth (0.3% beef extract, 0.5% peptone) (Fisher Scientific) and grown at 28°C for 24 h and shaken at 150 rpm. This condition is required for *Xanthomonas citri* subsp. *citri* to reach the log phase, a stage at which the bacterium is actively growing. The bacterial suspension was centrifuged at 5,000 × *g* for 10 min at 4°C, re-suspended in sterile saline phosphate buffer (PBS; 40 mM Na_2_HPO_4_ + 25 mM KH_2_PO_4_) and kept on ice. The bacterial suspension was adjusted to 0.3 OD at 600 nm, equivalent to 10^8^ colony-forming units (cfu) mL^-1^ for gene expression studies. For disease resistance screening, the suspension was adjusted to 10^4^ cfu mL^-1^. To confirm viability of the bacterium, the suspension was serially diluted and 50 μL of the final dilution was spread on each of two nutrient agar plates.

**Table 2:** q-PCR primer sequences of the selected defense genes and internal control genes.

Gene	Gene ID	Gene annotation	Forward 5′–3′	Reverse 5′–3′
**Internal control genes**		
FBOX	102626717	F-box/kelch-repeat protein	TTGGAAACTCTTTCGCCACT	CAGCAACAAAATACCCGTCT
GAPC2	837904	Glyceraldehyde-3-phosphate dehydrogenase	TCTTGCCTGCTTTGAATGGA	TGTGAGGTCAACCACTGCGACAT
**Target genes**		
CAO	818849	Copper amine oxidase	AACACTTCTTCATTGCCCGT	GTTCTCGTATTCCTCACAATCCAG
APX	107826841	Ascorbate oxidase	AGCAGTTCCCTACCATCTCC	CTCAGCCTTGTCATCTCTTCC
CAT	18641273	Catalase	GGACCCAACTACTTGATGCT	GTAATCGACCTCCTCATCCC
COI	732662	Coronatine-insensitive	GGCTCCTTCAATCATCCACC	ACATTCCTCGTCTCAAGAATTTCC
LOB	102614340	Lateral organ boundaries	TCCACCAACCGAACCATACA	GGCACTTGCTTCATAGACCAT
OPR2	102631049	12-Oxophytodienoate reductase 2	GGGCAGCAAACTGTGAGGAC	CAGATAGGGTTGGGATAAG
ICS1	102630235	Isochorismate synthase	GGAGGAGGAGAGAGTGAATTTG	GGGTTGCTTCCTTCTACTATCC
PAL	102620173	Phenylalanine ammonia lyase	CACATTCTTGGTAGCGCTTTG	AGCTACTTGGCTGACAGTATTC
NPR1	102617188	Non-expressor of PR genes 1	AACTCGCCTCAAGACTACCT	TGCAACTGTGTCGTTCCATA
PR2	102623685	β-1,3-Glucanases	TTCCACTGCCATCGAAACTG	TGTAATCTTGTTTAAATGAGCCTCTTG
PR5	102621661	Thaumatin-like protein	TACCTCCACCTCTCTCATTCTT	GTGCGAGAGAAGGTTAGCTATG


### Attached Leaf Assay

The attached leaf assay was conducted with the genotypes listed in Table 1. The plants were pruned to stimulate the growth of new flushes. The most susceptible leaf stage for citrus canker inoculation was reached 2–3 weeks after pruning when immature leaves were 75% expanded. Bacterial suspension was pressure infiltrated in young leaves using a 1 cm^3^ needle-less tuberculin syringe. The syringe tip was pressed against the abaxial surface of the leaf. The bacterial suspension (approximately 2 μL) was infiltrated into the leaf until the water-soaked area reached about 6 mm in diameter (Francis et al., 2010). Three areas on each side of the leaf mid-vein were infiltrated. Excess inoculum was wiped from the leaf surface with KIMTECH delicate task wipes (Kimberly-Clark, Koblenz, Germany). Six leaves per plant and six plants per genotype were inoculated. The inoculated flushes were covered with clear plastic bags for 24 h to maintain high humidity. The plants were completely randomized and kept in the greenhouse at a temperature range from 20 to 30°C. Leaves inoculated with sterile PBS buffer were used as the control following the same protocol for infiltration and observation. Development of symptoms on leaves was observed weekly up to 21 days post inoculation (dpi). The number of lesions per leaf was counted at 14 dpi. The experiment was repeated three times. The sum of number of lesions per leaf was analyzed using the General Linear Models procedure (SAS Institute, Cary, NC, United States). Mean separation was performed according to the Student–Newman–Keuls test (*P* ≤ 0.05).

### Bacterial Growth Curve *in vivo*

Bacterial growth curves were performed on selected genotypes. The genotypes chosen for comparison of resistance to *Xanthomonas citri* subsp. *citri* were ‘Meiwa’ kumquat, ‘Marsh’ grapefruit and two cybrids, M-9 (Cybrid-K type) and M-102 (Cybrid-G type). The leaves were inoculated as described previously in the attached leaf assay (Francis et al., 2010). Three 1-cm leaf disks per genotype, circumscribing the infiltrated area, were excised using a 1-cm cork borer immediately after inoculation and then at days 3, 8, 10, and 14. Leaf disks were surface disinfested by dipping in 0.5% sodium hypochlorite for 1 min, followed by immersion in 70% ethanol for 1 min and rinsed twice with sterile distilled water. Each disk was macerated individually in 1.0 mL of PBS buffer using a sterile mortar and pestle under aseptic conditions. Tenfold serial dilutions of the leaf suspension were made and dilutions were plated in duplicate on nutrient agar medium. One disk per plant and three plants per genotype were assayed for each time point. Total bacterial colonies per inoculation site were expressed as log_10_ cfu per inoculation site. The experiment was repeated three times. The log transformation of bacterial population per inoculation sites was analyzed using the ANOVA procedure (SAS Institute). Mean separation was performed according to Tukey’s Studentized Range (HSD) Test (*P* ≤ 0.05).

### Total RNA Isolation

To identify genes that were differentially expressed between the four genotypes, a time-course experiment was designed utilizing the citrus/*Xanthomonas citri* subsp. *citri* pathosystem. Five *Xanthomonas citri* subsp. *citri* or mock-inoculated plants of each genotype were used and each plant was considered an independent biological replicate. Attached leaves were washed with distilled water and followed by pressure infiltration inoculation on the abaxial side using a 1 cm^3^ needleless tuberculin syringe. Three inoculations, each composed of 2 μL of bacterial suspension, were made on both sides of the mid-vein. Six 1-cm leaf disks per leaf, circumscribing the infiltrated area, were excised using a 1-cm cork borer. The disks were placed in sterile RNase-free 2-mL tube, immediately immersed in liquid nitrogen and stored at -80°C until RNA extraction. Leaves inoculated with sterile PBS buffer were used as the control following the same protocol for inoculation and sample collection.

Leaf disks were harvested from untreated, inoculated and mock-inoculated plants before inoculation (time point 0) and 4, 24, and 96 h after inoculation. Leaf disks (100 mg) after freezing in liquid nitrogen were ground using Tissue Lyser II system (Qiagen, Valencia, CA, United States) for disruption and homogenization of leaf tissue through high-speed shaking in 2-mL plastic tubes containing one stainless steel bead. The tubes were shaken twice at 30 rps for 30 s. Total RNA was isolated from macerated leaf samples using RNeasy Plant Mini Kit (Qiagen) according to the manufacturer’s recommendations. The RNA samples were treated with RNase-free DNAse (Qiagen) to remove any contaminant genomic DNA. To ensure the quality of RNA samples, RNA concentration and integrity was measured by NanoDrop^®^ spectrophotometer, 2100 Bioanalyzer (Agilent Technologies, Palo Alto, CA, United States) and bleach agarose gel (Aranda et al., 2012).

### Quantitative Polymerase Chain Reaction (qPCR)

Independent RNA samples extracted from five untreated plants, five plants inoculated with *Xanthomonas citri* subsp. *citri* and five plants inoculated with PBS buffer for each genotype (‘Marsh’ grapefruit, ‘Meiwa’ kumquat, M-9 cybrid and M-102 cybrid) per each time point (0, 4, 24, and 96 hai) were used for quantitative PCR. From the RNA isolated as described above, cDNAs were synthesized from 1 μg of total RNA using QuantiTect Reverse Transcription Kit (Qiagen, Valencia, CA, United States) following the manufacturer’s instructions.

Quantitative PCR was performed using QuantiTech SYBR^®^Green PCR Kit (Qiagen). The reaction consisted of 1.0 μL of cDNA and 500 nM of each gene-specific primer in a final volume of 20 μL. Amplification was carried out for two technical replicates for each sample, including negative controls. Reactions were performed in a ABI Prism7500 sequence detector (Applied Biosystems, Foster City, CA, United States) with the following thermal cycles: 50°C for 2 min, 95°C for 10 min; 40 cycles of 95°C for 15 s, and 60°C for 1 min. Expression levels were assessed based on the number of amplification cycles needed to reach a fixed threshold (cycle threshold – Ct) in the exponential phase of PCR. Ct data were analyzed using the 7500 software v.2.0.6. (Applied Biosystems). For relative quantification, change in gene expression was calculated by the ΔΔCt method (Livak and Schmittgen, 2001) comparing both *Xanthomonas citri* subsp. *citri*-inoculated samples and mock-inoculated controls to non-inoculated sample for each time point. The relative expression is presented as log_2_-fold change.

**Table 3:** Mean of total number of citrus canker lesions on attached leaves in three independent experiments on three grapefruit cultivars, 22 cybrid clones and ‘Meiwa’ kumquat inoculated with *Xanthomonas citri* subsp. *citri* by using the pressure infiltration method.

Genotype	Mean of total number of lesion per leaf		
	
	Experiment 1^x^	Experiment 2^x^	Experiment 3^x^
‘Flame’ grapefruit	-^y^	138.16*a*	111.13*b*
‘Marsh’ grapefruit	129.25*a*	104.73*b*	119.93*a*
N-4*z*	86.620*c*	103.10*b*	77.63*d*
‘Ruby Red’ grapefruit	-	101.70*b*	90.36*c*
M-102*z*	109.92*b*	99.36*b*	96.96*c*
M-78*z*	106.33*b*	98.13*b*	90.33*c*
M-81*z*	-	98.03*b*	89.66*c*
M-13	60.25*fgh*	85.26*b*	57.60*ghi*
M-31	83.33*cd*	78.53*c*	-
N-10	73.62*def*	78.36*cd*	52.90*hi*
N-8	57.00*ghi*	76.13*cd*	-
N-18	53.66*hi*	72.70*cd*	57.06*ghi*
F-2	73.37*def*	70.23*de*	65.00*efg*
F-5	70.87*defg*	64.33*ef*	56.62*ghi*
M-10	-	63.26*efg*	58.73*fghi*
F-13	-	61.86*efg*	51.33*i*
F-3	53.25*hi*	58.13*fgh*	65.83*efg*
M-11	52.08*hi*	56.73*fgh*	62.06*fghi*
F-15	-	55.83*fgh*	57.96*ghi*
M-9	46.41*i*	53.90*fgh*	58.60*fghi*
N-6	63.58*fgh*	50.93*gh*	74.43*de*
F-10	64.62*efgh*	48.56*h*	70.03*def*
F-6	78.16*cde*	46.10*h*	64.30*efgh*
N-12	70.50*defg*	-	65.36*efg*
F-20	65.79*efgh*	-	-
‘Meiwa’ kumquat	1.20*j*	3.86*i*	22.23*j*


*The number of lesions was counted 14 days post-inoculation and six plants per genotype were inoculated.**x**Means in the same column followed by different letters are significantly different based on Student-Newman–Keuls test (α = 0.05).**y**Missing values. Some genotypes did not grow young vegetative flushes in time for inoculation.**z**Cybrids with grapefruit chloroplast (Cybrid-G).*

Primers specific to 10 selected genes were designed using the PerlPrimer software (Marshall, 2004) based on the mRNA sequences (Table 2). GAPC2 and FBOX were selected as internal reference genes (Mafra et al., 2012; Yan et al., 2012). The sequences of endogenous gene primers are listed in Table 2. For a trustworthy analysis, relative quantitative methods assume that the target and endogenous genes amplify with similar efficiency. The amplification rate was calculated on the basis of a linear regression slope of a dilution row and efficiency (*E*) was determined based on the equation *E* = 10^[-1/slope]^ (Pfaffl, 2002).

**FIGURE 1:**
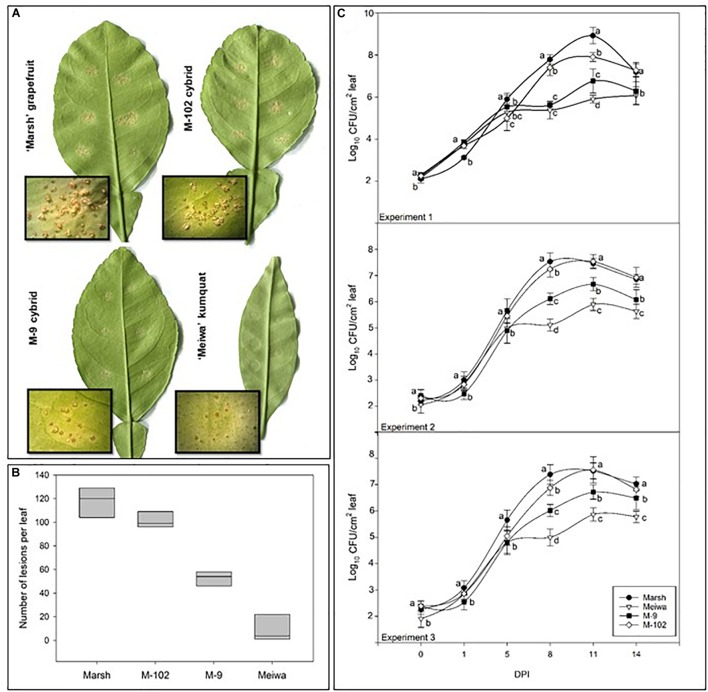
Characterization of citrus canker resistance in grapefruit cybrids. **(A)** Citrus canker lesions in ‘Marsh’ grapefruit, ‘Meiwa’ kumquat, cybrid M-9 and cybrid M-102. Photos were taken 14 days post-inoculation with *Xanthomonas citri* subsp. *citri* suspension at 10^4^ cfu mL^-1^. **(B)** Mean of total number of lesions per leaf on attached leaves of ‘Marsh’ grapefruit, ‘Meiwa’ kumquat, as well as the cybrids M-9 and M-102 14 days after pressure infiltration inoculation of *Xanthomonas citri* subsp. *citri* suspension at 10^4^ cfu mL^-1^. Data represent the average of six leaves from five biological replicates with SD. **(C)** Growth curves for *Xanthomonas citri* subsp. *citri* on attached leaves of ‘Marsh’ grapefruit, ‘Meiwa’ kumquat, cybrid M-9 and cybrid M-102 during 14-day period after pressure infiltration inoculation with *Xanthomonas citri* subsp. *citri* suspension at 10^4^ cfu mL^-1^. *Xanthomonas citri* subsp. *citri* population was estimated by counting the number of *Xanthomonas citri* subsp. *citri* colonies and expressed as log_10_ of bacteria per leaf per time point. Data followed by the same letter for each time point are not significantly different at *P* ≤ 0.05 according to Tukey’s Studentized Range (HSD) test. The experiment was repeated three times with similar results.

## Results

### Citrus Canker Response in Grapefruit Cybrids

To examine the effect of cybridization on canker response, 22 independently derived cybrids (4 Cybrid-G and 18 Cybrid-K) along with grapefruit and kumquat controls were examined for response to the pathogen by an attached leaf assay. Citrus canker symptoms appeared on grapefruit cultivars about 7 days post inoculation (dpi) with a suspension of *Xanthomonas citri* subsp. *citri* at 10^4^ cfu mL^-1^. The lesions were characterized by raised, light brown, blister-like pustules typical of the compatible host reaction (Figure 1A). Cybrids showed the same typical canker lesions; however, the number of lesions varied (Figure 1B). Most of the ‘Meiwa’ kumquat leaves did not show any symptoms, but a few inoculation sites showed slightly raised, dark brown pustules (Figure 1A). No sign of a hypersensitive response (HR) was observed.

All cybrids with kumquat chloroplasts (Cybrid-K) had significantly fewer lesions (46–85 lesions per leaf), compared to their grapefruit parent cultivars (Table 3). ‘Marsh,’ ‘Flame,’ and ‘Ruby Red’ grapefruit cultivars had consistently higher numbers of lesions in all experiments (>104 lesions per leaf) (Table 3 and Figure 1B). ‘Flame’ grapefruit had the highest number of lesions (138 lesions per leaf). Cybrids with grapefruit chloroplasts (Cybrid-G) had the highest number of lesions among all cybrids (86–109 lesions per leaf) and did not significantly differ in number of lesions compared to ‘Ruby Red’ grapefruit in Experiment 2 or 3 (Table 3). ‘Meiwa’ kumquat had the lowest number of lesions in all experiments (1–20 lesions per leaf) (Table 3 and Figure 1B). Based on performance in the attached leaf assay, two cybrids were selected for detailed studies: M-102 (a Cybrid-G type) and M-9 (a Cybrid-K type).

Bacterial growth curves were examined in three independent experiments (Figure 1C). Around 100 cfu of *Xanthomonas citri* subsp. *citri* were detected from leaf disks of all genotypes immediately after inoculation. All genotypes showed similar bacterial growth until 5 dpi (∼10^5^ cfu). At 8 and 11 dpi, significant differences in bacterial populations were observed among the genotypes. ‘Marsh’ grapefruit showed a rapid increase in *Xanthomonas citri* subsp. *citri* multiplication, reaching 10^8^ cfu per disk at 11 dpi. ‘Meiwa’ kumquat showed the lowest bacteria population of 10^5^ cfu per disk at 14 dpi. Interestingly, the cybrids followed the same bacterial growth pattern as their chloroplast donor parent. Bacteria multiplied rapidly in the M-102 cybrid, which carries grapefruit chloroplasts, and at 5 dpi in experiments 2 and 3, the *Xanthomonas citri* subsp. *citri* populations did not significantly differ from those of ‘Marsh’ grapefruit. In all experiments, the M-9 cybrid with kumquat chloroplasts had populations one to two log units lower than ‘Marsh’ grapefruit at 11 dpi. At 14 dpi, *Xanthomonas citri* subsp. *citri* populations declined in all genotypes (Figure 1C).

**FIGURE 2:**
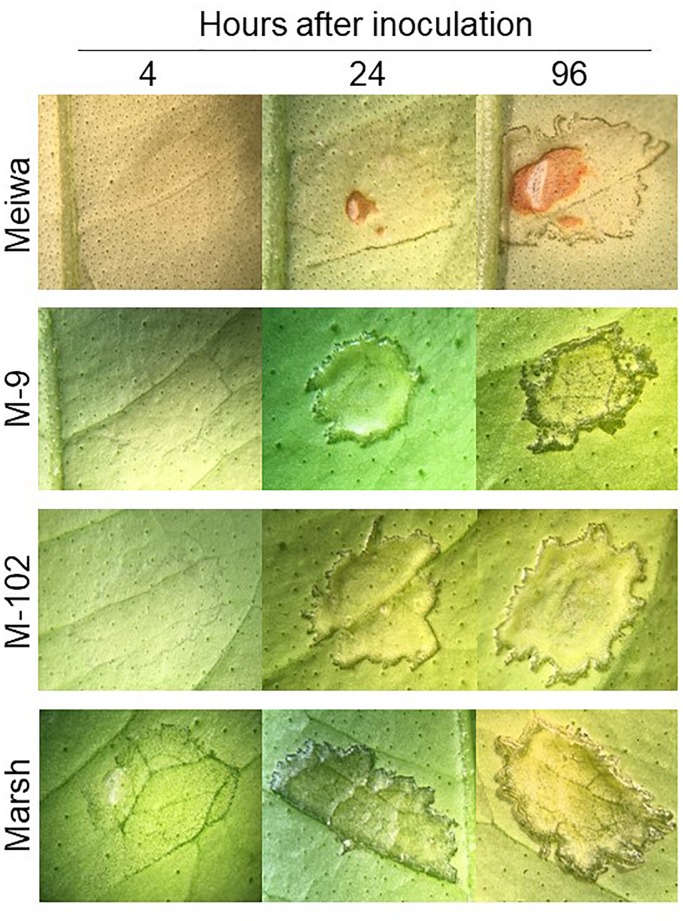
Citrus canker symptoms after pressure infiltration with *Xanthomonas citri* subsp. *citri* suspension at 10^8^ cfu mL^-1^. Comparison of canker disease development in leaves of ‘Meiwa’ kumquat, M-9 cybrid, M-102 cybrid and ‘Marsh’ grapefruit after pressure infiltration with *Xanthomonas citri* subsp. *citri* suspension at high concentration. Pictures were taken at 4, 24, and 96 hours post inoculation (hpi).

**FIGURE 3:**
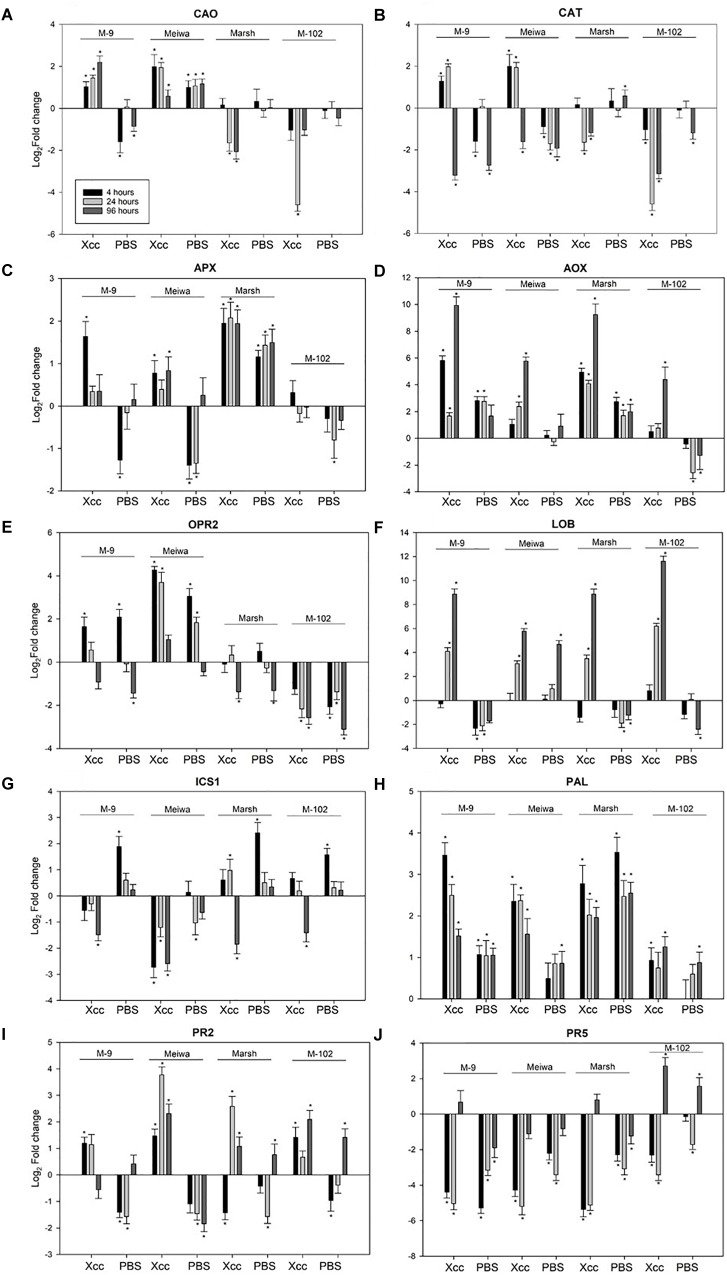
Expression profiles of ROS, JA, SA-related genes and *PR* genes in ‘Meiwa’ kumquat, M-9 cybrid, M-102 cybrid and ‘Marsh’ grapefruit in response to *Xanthomonas citri* subsp. *citri* inoculation and mock-treatment with PBS buffer. Each graphic represents one gene and each bar represents one time point. Copper amine oxide **(A)**, catalase **(B)**, ascorbate peroxidase **(C)**, alternative oxidase **(D)**, 12-oxophytodienoate reductase 2 **(E)**, and lateral organ boundaries **(F)**, isochorismate synthase **(G)**, phenylalanine ammonia lyase **(H)**, endo-β-1,3-glucanases **(I)** and thaumatin protein **(J)**. Gene expression columns followed by an asterisk are significant at *P* ≤ 0.05 according to Student’s *t*-test.

### Gene Expression Features of Cybrid and Parent Genotypes

To gain insight into the role of chloroplasts in plant defense, expression of some defense-related genes in response to *Xanthomonas citri* subsp. *citri* inoculation was analyzed. To identify differences in the *Xanthomonas citri* subsp. *citri* response between ‘Meiwa’ kumquat, ‘Marsh’ grapefruit, M-9 cybrid (Cybrid-K) and M-102 cybrid (Cybrid-G), leaves of these four genotypes were pressure infiltrated with *Xanthomonas citri* subsp. *citri* at 10^8^ cfu mL^-1^ and kept under greenhouse conditions. At 4 hours post inoculation (hpi), only ‘Marsh’ leaves started to show water-soaking symptoms with the darkening of the inoculation site (Figure 2). At 24 hpi, the water-soaking progressed to tissue hyperplasia in ‘Marsh’ as well as M-9 and M-102 cybrids, whereas ‘Meiwa’ kumquat showed a slight water-soaking and necrosis at the center of the inoculation site. At 96 hpi, ‘Marsh’ grapefruit and the cybrids M-9 and M-102 showed similar hyperplasia and hypertrophy symptoms typical of canker lesions, while in ‘Meiwa’ kumquat, the necrotic spot resembled an HR (Figure 2). Thus, although M-9 was exhibited resistance to citrus canker when inoculated with *Xanthomonas citri* subsp. *citri* at 10^4^ cfu mL^-1^, under high bacteria concentration ‘Marsh’ and M-9 expressed a similar compatible interaction, while ‘Meiwa’ showed signs of necrosis, corresponding to an incompatible interaction.

The four genotypes differed not only with respect to their inoculation phenotypes. The expression levels of selected defense-related genes were significantly different among the four genotypes evaluated. These 10 genes were divided into sub-categories according to their function. Among genes related to reactive oxygen species (ROS) pathways, *copper amine oxidase* (*CAO*) was upregulated in ‘Meiwa’ and M-9 cybrid at all time points after *Xanthomonas citri* subsp. *citri* inoculation. Under the same conditions, *CAO* was downregulated in ‘Marsh’ and M-102 cybrid (Figure 3A). Similar results were observed for *catalase* (*CAT*). *CAT* was upregulated in ‘Meiwa’ kumquat and M-9 cybrid and downregulated in ‘Marsh’ and M-102 cybrid in the first 24 h (Figure 3B). *Ascorbate peroxidase* (*APX*) was upregulated in all genotypes, except for the M-102 cybrid, but with a higher expression in ‘Marsh’ (Figure 3C). However, *APX* was also upregulated in ‘Marsh’ treated with PBS buffer. Alternative oxidase (AOX) is known to be associated with mitochondrial ROS production. Both cybrids have the same nuclear and mitochondrial genotypes, but they showed different *AOX* expression patterns. The M-9 cybrid and ‘Marsh’ grapefruit share the same nuclear genotype and have different mitochondrial genotypes, but *AOX* expression was similar in these genotypes (Figure 3D).

The *12-oxophytodienoate reductase 2* (*OPR2*) gene, encoding a key enzyme for jasmonic acid (JA) synthesis, was upregulated in canker-resistant genotype ‘Meiwa’ kumquat at all time points after *Xanthomonas citri* subsp. *citri* inoculation (Figure 3E). This upregulation was also observed in the canker moderate resistant M-9 cybrid at 4 and 24 hpi, but absent or downregulated in susceptible ‘Marsh’ grapefruit and the M-102 cybrid. In ‘Marsh,’ *OPR2* was downregulated at 96 hpi, and in M-102 cybrid, it was downregulated at all time points. However, in all genotypes, both *Xanthomonas citri* subsp. *citri* and mock treatments induced similar expression pattern for this gene. The *lateral organ boundaries* (*LOB*) gene, encoding a transcription factor that acts as a repressor of JA-regulated defense genes, was upregulated in all genotypes at 24 and 96 hpi (Figure 3F). After PBS treatment, *LOB* was upregulated in ‘Meiwa’ while it was downregulated in the other genotypes.

Genes encoding the enzymes responsible for salicylic acid (SA) synthesis, *isochorismate synthase* (*ICS1*) and *phenylalanine ammonia lyase* (*PAL*) were also evaluated. Overall, ‘Meiwa’ and the M-9 cybrid showed a downregulation of *ICS1* whereas ‘Marsh’ and the M-102 cybrid showed an upregulation after *Xanthomonas citri* subsp. *citri* inoculation (Figure 3G). After mock inoculation treatment, *ICS1* was upregulated in ‘Marsh’ grapefruit and both cybrids. *PAL* was upregulated in all genotypes at all time points under both conditions, but ‘Meiwa’ and the M-9 cybrid showed a higher expression of *PAL* when treated with *Xanthomonas citri* subsp. *citri* compared to water (Figure 3H).

*Pathogenesis-related* (*PR*) genes encoding endo-β-1,3-glucanase (*PR-2*) and thaumatin protein (*PR-5*) were investigated (Figures 3I,J). *PR-2* was upregulated in ‘Meiwa’ at all-time points, showing a high expression at 24 hpi. ‘Marsh’ grapefruit showed an upregulation of this gene only at 24 hpi. In both cybrids, *PR-2* was upregulated under *Xanthomonas citri* subsp. *citri* inoculation. In contrast, *PR-5* was downregulated in all genotypes under both treatment conditions with the exception of 96 hpi in M-102, a time point at which this gene was upregulated.

**FIGURE 4:**
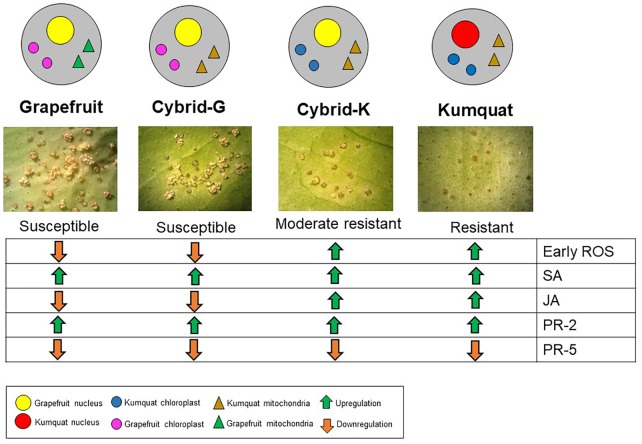
Gene expression overview of ‘Marsh’ grapefruit, M-102 cybrid (Cybrid-G), M-9 cybrid (Cybrid-K) and ‘Meiwa’ kumquat in response to *Xanthomonas citri* subsp. *citri* infection. Green arrows indicate upregulation and orange arrows indicate downregulation.

## Discussion

The hypothesis of this study is that kumquat chloroplasts and/or mitochondria, when combined with the grapefruit nucleus, may enhance grapefruit resistance to citrus canker. Kumquats are considered highly resistant to citrus canker (Khalaf et al., 2007). Although this species is in a different genus than commercial citrus, kumquats can be used to study inheritance of canker resistance due to their sexual compatibility with citrus (Khalaf et al., 2007). Kumquat resistance to citrus canker has been characterized as an HR reaction to *Xanthomonas citri* subsp. *citri* infection, which blocks the bacterial proliferation in the plants (Trivedi and Wang, 2014). The new combinations of kumquat organelles and grapefruit nucleus opened a door to investigate the influence of the kumquat chloroplasts and/or mitochondria in citrus canker response (Omar et al., 2017).

Attached leaf assays by pressure infiltration inoculation with *Xanthomonas citri* subsp. *citri* suspension of 10^4^ cfu mL^-1^ enable differentiation of citrus genotype response based on the number of disease lesions (Francis et al., 2009). Our results (Figure 1) confirmed that ‘Marsh,’ ‘Flame,’ and ‘Ruby Red’ grapefruit are highly susceptible to citrus canker (Gottwald et al., 2002), because these cultivars produced raised blister-like leaf lesions typical of a compatible interaction. ‘Meiwa’ kumquat showed contrasting reaction to *Xanthomonas citri* subsp. *citri* inoculation and no canker lesions were observed on most of the inoculated leaves. Few leaves showed slightly raised dark brown lesions at the inoculation site; but no sign of an HR reaction was observed. Grapefruits had an average of 117 lesions per leaf compared to nine lesions observed in kumquat based on three independent experiments (Table 3). In addition, at 8 dpi, the bacterial population in the kumquat leaves was about 100-fold less than grapefruit leaves (Figure 1C). These results substantiate the use of grapefruit cultivars and ‘Meiwa’ kumquat as susceptible and resistant controls, respectively.

The response of grapefruit cybrids to *Xanthomonas citri* subsp. *citri* inoculation was previously unknown. The attached leaf assay was further used to investigate the reaction of four independent Cybrid-G and 18 independent Cybrid-K clones to citrus canker. The total number of lesions per inoculation site varied among the cybrids; however, all Cybrid-K types had a significantly lower number of lesions compared to grapefruit cultivars and to Cybrid-G types (Table 3 and Figure 1B). In the genetic background comprised of the grapefruit nuclear and mitochondrial genotypes, the kumquat chloroplast genotype clearly co-segregated with increased resistance to *Xanthomonas citri* subsp. *citri*.

In addition, the bacterial populations in a selected Cybrid-K (M-9) were 10- to 100-fold lower than those in the ‘Marsh’ grapefruit or in a selected Cybrid-G (M-102) (Figure 1C). These results indicate that even though both cybrids have the same grapefruit nucleus and mitochondria, their responses against artificial *Xanthomonas citri* subsp. *citri* inoculation were significantly different. Cybrids with kumquat chloroplasts (Cybrid-K) were more resistant to citrus canker than cybrids with grapefruit chloroplasts (Cybrid-G) with respect to both lesion number and bacteria multiplication (Table 3 and Figure 1C). These data clearly demonstrate the role of kumquat chloroplasts in the reduction of citrus canker severity in grapefruit cybrids, since the only genetic difference between both cybrids is the chloroplast genome. These findings demonstrate an important role of chloroplast in the resistance of citrus cybrids to citrus canker.

Several studies have demonstrated the role of chloroplasts in plant resistance against biotic stress, but most of these experiments were done in *Arabidopsis thaliana* or tobacco (*Nicotiana benthamiana*) (Nomura et al., 2012; De Torres Zabala et al., 2015; Serrano et al., 2016). So far, no research on perennial trees such as citrus has been conducted to establish the function of chloroplasts in disease resistance. Chloroplasts are vital organelles for plants and are well known to be an active metabolic center by converting solar energy to carbohydrates through the process of photosynthesis (Daniell et al., 2016). In addition to this primary function, chloroplasts play a role in other vital physiological processes in plants including synthesis of amino acids, nucleotides and vitamins. Chloroplasts also host a variety of intermediate metabolic pathways, such as biosynthesis of plant hormones including SA, JA, abscisic acid (ABA), indole-3-acetic acid (IAA), and cytokinins (CKs) and generation of ROS (Nomura et al., 2012; Pfannschmidt and Yang, 2012). Therefore, chloroplasts have a central role in sensing and signaling cellular responses to various stimuli (Pfannschmidt and Yang, 2012), including pathogen attack.

To further investigate how plastid genotype might affect the *Xanthomonas citri* subsp. *citri* response, quantitative PCR analysis targeting chloroplast-related genes was performed to compare the gene expression of four genotypes, canker susceptible ‘Marsh’ grapefruit and M-102 cybrid (Cybrid-G type), canker moderate resistant M-9 cybrid (Cybrid-K type) and canker resistant ‘Meiwa’ kumquat. M-9 and M-102 cybrids showed a strong contrasting response to *Xanthomonas citri* subsp. *citri* inoculation (Table 3). These genotypes were chosen to test candidate genes for possible involvement in the differential pathogen response. Because only one cybrid of each type was tested, the responses identified and discussed below must be further tested to confirm their association with disease phenotypes.

Reactive oxygen species generated in chloroplasts is responsible for triggering signaling pathways that influence nuclear encoded genes (Balazadeh et al., 2012). Hydrogen peroxide (H_2_O_2_) is produced in response to a variety of different stress conditions, including biotic stress, and is scavenged by different antioxidant/enzyme reactions in the ascorbate and glutathione cycles involving APX, catalase (CAT), and peroxiredoxin (PRX) (Tripathi et al., 2009). CAO catalyzes the degradation of cellular polyamines, producing H_2_O_2_ derived from polyamine oxidation. This process has been correlated with wound-healing and cell wall reinforcement during pathogen invasion (Liu et al., 2015). Notably, both *CAO* and *CAT* were upregulated in M-9 and ‘Meiwa’ and downregulated in M-102 and ‘Marsh’ at 4 and 24 hpi (Figures 3A,B). These results indicate a clear correlation among the ROS gene expression, canker resistance, and chloroplasts in the ‘Meiwa’ kumquat and M-9 cybrid in ROS gene expression, suggesting that ROS pathway in citrus depends on the chloroplast since ‘Meiwa’ and M-9 share the same chloroplast genome. Previous studies have shown that expression profiles of genes related to ROS differ in resistant and susceptible genotypes under biotic stress (Coram and Pang, 2006; Jain et al., 2016); however, this is the first time that this function has been linked to the chloroplast alone. The gene expression of *APX* was similar in the M-9 cybrid and ‘Meiwa’ where the gene is upregulated only after *Xanthomonas citri* subsp. *citri* inoculation. On the other hand, in ‘Marsh,’ *APX* is upregulated after *Xanthomonas citri* subsp. *citri* and water infiltration of leaves, suggesting that the activation of ROS in the susceptible genotype is a response to wounding and not specifically to *Xanthomonas citri* subsp. *citri*.

We also evaluated expression of the gene encoding mitochondrial AOX, proposed to prevent ROS formation when the cytochrome respiration pathway is compromised. In citrus plants, AOX is described as having an important role during biotic stress in non-host plant pathogen interaction (Daurelio et al., 2009). Kumquat and all cybrids have the same mitochondrial genotype but *AOX* showed a different gene expression profile for each genotype (Figure 3D). This contrasting expression between the cybrids can be explained by the cross talk of mitochondria and chloroplasts. Even though both organelles have been considered autonomous, they are not completely independent and researches have shown several metabolic, functional and physical inter-connections between mitochondria and chloroplasts (Shapiguzov et al., 2012; Trotta et al., 2014). The M-102 cybrid is the only genotype that has chloroplast (grapefruit) and mitochondria (kumquat) from different parents and this new combination could have caused a change in the functioning of both organelles.

Salicylic acid and JA are phytohormones that play an important role in different signaling pathways involved in plant defense against a variety of biotic and abiotic stresses (Hu et al., 2009; Lu, 2009; War et al., 2011). SA is synthesized in chloroplasts from chorismic acid by ICS1 (Serrano et al., 2013), but can also be synthesized from phenylalanine by PAL (Kim and Hwang, 2014). Our results suggest that, after pathogen attack, early biosynthesis of SA is controlled only by PAL in the M-9 cybrid and ‘Meiwa’ kumquat, because the expression of *ICS1* was downregulated after *Xanthomonas citri* subsp. *citri* inoculation of both genotypes (Figure 3H). On the other hand, in ‘Marsh’ grapefruit, genes of both SA pathways were upregulated, but the higher expression in leaves inoculated with PBS suggests that SA is not used as a defense signaling pathway in this susceptible genotype. For the M-9 cybrid and ‘Meiwa,’ the early chloroplast-dependent SA biosynthesis by PAL may be involved in the resistance to *Xanthomonas citri* subsp. *citri* since the upregulation of this gene was higher after *Xanthomonas citri* subsp. *citri* inoculation than mock inoculation (Figure 3H). PAL has been demonstrated to be required for the induction of SA-dependent signaling in pepper defense against *Xanthomonas campestris* pv. *vesicatoria* (Kim and Hwang, 2014).

Jasmonic acid is a plant hormone whose biosynthesis begins in the chloroplast but is completed in the peroxisome (Serrano et al., 2016). The enzyme 12-oxophytodienoate reductase 2 (OPR2) is involved in the biosynthesis of JA. ‘Meiwa’ kumquat and M-9 cybrids showed a contrasting *OPR2* gene expression compared to ‘Marsh’ grapefruit and M-102 cybrids (Figure 3E). *OPR2* was induced by both *Xanthomonas citri* subsp. *citri* and mock inoculation at 4 hpi in the ‘Meiwa’ kumquat and the M-9 cybrid, but was not expressed or downregulated in the ‘Marsh’ grapefruit and M-102 cybrid. Therefore, biosynthesis of JA appears to be important for citrus defense against *Xanthomonas citri* subsp. *citri*, but it is not *Xanthomonas citri* subsp. *citri* specific. Another gene evaluated encodes the transcription factor LOB. This gene represses a subset of jasmonate-mediated defenses and is considered a critical canker disease susceptibility gene in citrus (Hu et al., 2014). *LOB* was upregulated in all genotypes at 24 and 96 hpi; however, in ‘Meiwa,’ this gene was upregulated after mock inoculation as well, while in the other genotypes, *LOB* was downregulated after mock inoculation (Figure 3F). This result suggests that the canker susceptibility gene *LOB* does not play a role in ‘Meiwa’ kumquat, which agrees with its resistant phenotype. In addition, *LOB* expression was higher in ‘Marsh’ and both cybrids at 96 hpi, a time point at which the bacteria are successfully multiplying inside the host.

Besides chloroplast-related genes, two defense-related genes were selected for qPCR. Production and accumulation of pathogenesis-related (PR) proteins in plants in response to the invading pathogen are very important for plant defense. PR proteins accumulate locally in the infected area and in remote uninfected tissues, where they can prevent the affected plants from further infection (Durrant and Dong, 2004). ‘Marsh’ grapefruit exhibited upregulation of *PR-2* at 24 hpi, however this gene activation did not prevent citrus canker development. Transcriptomic analysis with kumquat and grapefruit showed that *PR-2* is upregulated in both susceptible and resistant genotypes after *Xanthomonas citri* subsp. *citri* infection, therefore this gene is considered a basic plant defense gene but not a key for citrus canker resistance (Khalaf et al., 2007; Fu et al., 2012). *PR-5* seems to not be involved in citrus canker host–parasite interaction since it was downregulated under all treatments (Figure 3J). The summary of our results is described in Figure 4.

Our results strongly support that kumquat chloroplast transfer to grapefruit cybrids enhanced resistance to citrus canker. Plastid-nuclear genome incompatibilities potentially elicit or enhance the defense responses uncovered here. The assembly and function of photosynthetic electron transfer complexes is achieved through interaction of plastid and nuclear genetic systems. Hundreds of nuclear genes mediate transcription, transcript stability, intron splicing, RNA editing, translation, protein complex assembly and protein turnover in the plastid (Barkan, 2011; Belcher et al., 2015; Ichinose and Sugita, 2016). The co-evolution of these interactions creates species-specific differences such that novel nuclear-plastid genome combinations can produce subtle or significant differences in plastid function (Herrmann et al., 2002; Greiner and Bock, 2013). Failure to correctly edit the *Arabidopsis thaliana* plastid *ndhD* transcript impairs cyclic electron flow around photosystem I with a concomitant increase in resistance to the fungal pathogen *Plectospherella cucumerina* (Garcia-Andrade et al., 2013). While the RNA editing defect was conditioned by a nuclear mutation in this example, the co-evolution of plastid RNA editing sites and their nuclear-encoded recognition factors is known to create species-specific editing requirements (Tillich et al., 2005). This might also be the situation for other types of plastid gene expression factors. The citrus cybrids and their parents described here lay the groundwork for mechanistic investigations of plastid signaling in plant defense. They raise the question of whether other alien plastid genomes, even those originating from susceptible genotypes, might condition reduced susceptibility to citrus canker and other diseases, leading to new avenues toward plant disease control.

It must be noted that all the canker assays discussed in this report were conducted in a greenhouse, and the improved response of the grapefruit cybrids containing kumquat chloroplasts against citrus canker must still be validated under real-world conditions in the field. Thus, the entire population of cybrid grapefruit plants, along with replicates of some of the more interesting clones, has been planted at a field site with heavy canker pressure near Fort Pierce, FL, United States. Citrus greening disease or huanglongbing (HLB) is also now endemic in this area and grapefruit is highly susceptible. It will be interesting to see if any of the cybrid grapefruit show a different response to HLB.

## Author Contributions

MM, AO, ZM, CC, JWG, and JHG contributed conception and design of the study. AO produced and regenerated all the cybrid materials used in this study. MM performed the experiments, statistical analyses, and wrote the first draft of the manuscript. All authors contributed to manuscript revision, read and approved the submitted version.

## Conflict of Interest Statement

The authors declare that the research was conducted in the absence of any commercial or financial relationships that could be construed as a potential conflict of interest. The reviewer FW declared a shared affiliation, with no collaboration, with several of the authors, ZM and CC, to the handling Editor at the time of review.
